# Supplementary material for: Accurate measurement of simulated slow and altered walking activity: Apple Watch best in class wearable devices
Source: PLoS One. 2025 Oct 22;20(10):e0333504. doi: 10.1371/journal.pone.0333504 (PMC12543184; doi:10.1371/journal.pone.0333504)
Supplement: S1 File — (DOCX) [file pone.0333504.s001.docx]

**Supplementary Material: Details of Devices**

(as at study inception – July 2019)

Table S1 – Study device costs and researcher* review comments


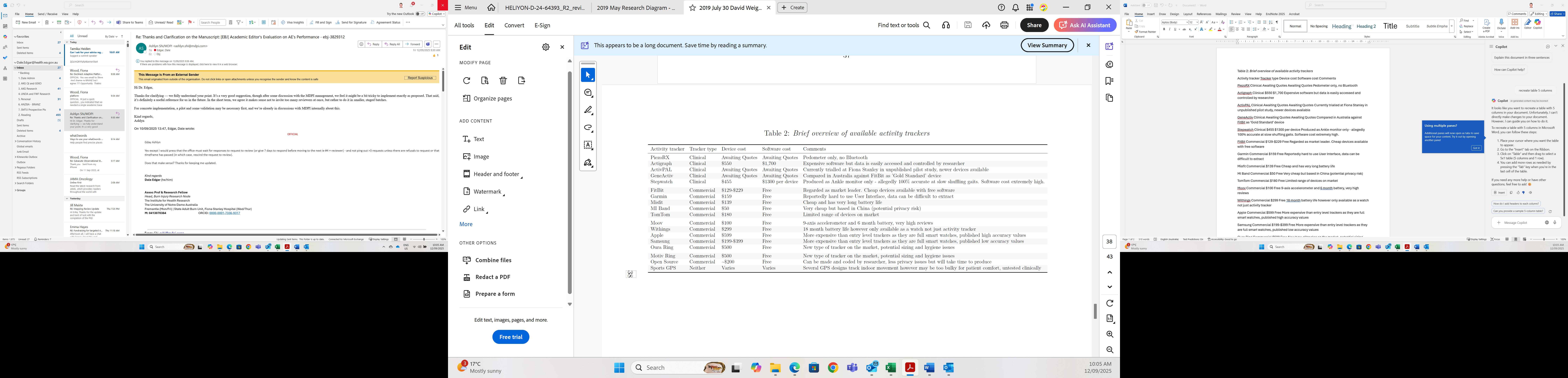


*Researcher comments provided by David Weight

Table S2 – Study device details

| **Study Device** | **Batch Manufacture Date** | **Tolerance Range** | **Calibration** |
| --- | --- | --- | --- |
| Samsung Galaxy Fit | 24/6/19 | Unavailable as these details are proprietary.  The study deployed devices 'not as intended' to test the limits of their capabilities. | All devices were deployed as standard ‘out of the box’ and were not subjected to any calibration. |
| Withings Steel HR | Q1/2 2018 |  |  |
| Garmin Vivofit 4 | Q1/2 2018 |  |  |
| Withings Move | Q1/2 2019 |  |  |
| Fitbit Charge 4 | April 2020 |  |  |
| Samsung Active 2 | 6/11/2019 |  |  |
| Garmin Forerunner 45S | April 2019 |  |  |
| Fitbit Inspire | Q1/2 2019 |  |  |
| Fitbit Versa 2^α^ | September 2019 |  |  |
| Actigraph WGT3X-BT | 2018 |  |  |
| GeneActiv | Company Uncontactable |  |  |
| Apple Watch Series 5 | Q1/2 2020 |  |  |

^α^ DW personal device
