# Supplementary material for: Accurate measurement of simulated slow and altered walking activity: Apple Watch best in class wearable devices
Source: PLoS One. 2025 Oct 22;20(10):e0333504. doi: 10.1371/journal.pone.0333504 (PMC12543184; doi:10.1371/journal.pone.0333504)
Supplement: Attachment — Submitted filename: wearables_PONE_Response to reviewers_R1_06082025.docx [file pone.0333504.s003.docx]

Response to reviewers: PONE-D-25-18604

We would like to thank the editor and reviewers for their thorough review of the manuscript and for their insightful comments. We have adjusted the study report and provide below our responses to each comment. We believe that the manuscript has been substantially improved by the changes we have made after consideration of suggestions. The editor and reviewer comments are reproduced in *italicized* font; our responses in regular, coloured font; revised or added sections of the manuscript are in red font; passages removed have been indicated by ~~strikethrough~~.

Lastly, noted page numbers refer to the revised manuscript document.

**EDITOR**

*Thank you for submitting your manuscript to PLOS ONE. After careful consideration, we feel that it has merit but does not fully meet PLOS ONE’s publication criteria as it currently stands. Therefore, we invite you to submit a revised version of the manuscript that addresses the points raised during the review process.*

We thank the editor for their encouraging comments.

**REVIEWER 1**

1. *Lack of real patient data: Although using healthy participants to simulate slow and shuffling gait is a reasonable starting point, the absence of clinical populations (e.g., elderly or neurologically impaired patients) limits the external validity. Simulated gait may not capture biomechanical and neuromuscular irregularities, such as asymmetrical patterns or compensatory strategies, that influence wearable device performance in actual hospital settings.*

We thank the reviewer for their comment and concur that generalisability of study results, is impacted. We have acknowledged that the use of healthy participants to simulate slow and shuffling gaits represents a limitation in *Strengths and Limitations*. However, we have now adjusted the study title and clarified statements in the Discussion. To provide context, some years before this study, we had observed in burn patients, using early movement sensor technology (now superseded), that gait speed, particular low gait speed, was strongly associated with missing step count data (statement added re activePAL study). Thus, when this study was devised, our primary aim was to: a) identify wearables that fell outside the accuracy parameters for our future research in the burn patient population; and, b) establish (test) a controlled framework to compare the accuracy of wearable activity monitors under standardised conditions, which allowed us to isolate device-related performance without the confounding variability inherent in clinical populations. We agree that simulated gait may not replicate the biomechanical and neuromuscular irregularities observed in clinical scenarios—such as asymmetries, compensatory strategies, or fatigue-related fluctuations—that can significantly influence step detection. We believe the existing text acknowledges the limitation but have added the following changes in the *Strengths and Limitations* section on page 18, which we believe helps highlight this issue with more clarity for readers.

Strengths and Limitations sub-section, page 20, changes from ~Line 482:

However, the study's focus on controlled conditions may limit ~~its~~ generalisability of findings to real-world applications, where variability in walking patterns, environmental factors, and patient-specific conditions is more pronounced, particularly in complex hospital settings. During preliminary testing using early generation movement sensors in a burn patient population, it was observed that reducing gait speed was associated strongly with increasing missing step count data (unpublished activPAL® data, Edgar DW, Chen A, Wood FW, 2012). Thus, to reduce confounders in this study, testing was conducted on self-reported healthy individuals who simulated altered, and specifically slow, walking patterns. It is further acknowledged, that while useful for preliminary evaluations, our ‘structured, reproducible’ movement adjustments may not fully replicate the biomechanical complexities of patients with clinical gait abnormalities. In addition, due to the significant time burden on participants to complete the study tasks, we did not include control testing with faster and, or ostensibly ‘normal’ walk speeds. While evidence exists evaluating device performance in clinical populations [48, 49], future research should build on this by further exploring their performance in specific groups, such as patients with trauma-induced gait impairments, neurological conditions, or post-surgical recovery [50]. These studies would provide more comprehensive insights into the devices' accuracy, reliability, and practical applications in real-world healthcare scenarios, particularly for monitoring and supporting patient rehabilitation.

2. *Underexplored device-specific limitations: The poor performance of devices like GENEActiv and Withings is reported, but not adequately analyzed. A more technical discussion is needed regarding the potential causes—such as accelerometer placement sensitivity, sampling frequency, lack of native step detection algorithms, or preprocessing methods—to provide useful guidance for both researchers and developers.*

We thank the reviewer for this query. As noted, post-processing procedures and assumptions likely played a central role in the observed performance differences, particularly for devices lacking native step detection algorithms. For example, devices such as GENEActiv required custom post-processing of raw acceleration data to estimate step counts, introducing unobserved confounding and potential additional sources of error not present in devices with integrated algorithms. While we provided a detailed explanation of our approach on page 7 (from ~line 166 - unchanged), we agree that further explanation of our methods is warranted and have added detail to the dedicated paragraph discussing these procedures on page 26. Accordingly, we have revised the manuscript to clarify the post-processing approaches used and to more explicitly acknowledge their potential contribution to device-specific variability in performance.

Discussion, page 16, changes from ~Line 368:

The GENEActiv device's poor performance in detecting slow or altered walking raises concerns about its suitability for hospital settings. Similarly, Mora-Gonzalez et al. (2022) found that at speeds of 0.8–1.6 km/h, the GENEActiv exhibited MAPE values of approximately 20 30%, highlighting difficulties in step detection during low-intensity activities [37, 38]. While these limitations may stem from the device’s sensor sensitivity and calibration—where oversampling during slow or altered movement could amplify noise and obscure true step events, further reducing accuracy—its poor performance in the present study likely reflects multiple factors, including also the data processing method used to determine step count. These findings underscore broader challenges clinicians and researchers face when using step-based metrics. Many step detection algorithms require predefined, often poorly defined, parameters, such as acceleration thresholds and activity mode, before processing data [39]. In the case of the GENEActiv device, the absence of native onboard step detection necessitates external data processing. In our study, as noted earlier, .bin files were analysed using the GENEAclassify package in R, applying the getGENEAsegments() function. This approach was intentionally selected as a simple, transparent, and reproducible method, designed to facilitate future uptake by clinicians and researchers who may not have advanced programming skills. Notably, a similar approach was employed by Schoenfelder et al. [1], who processed GENEActiv .bin files using the GENEAclassify package (v1.5.1) to derive stepping metrics from raw accelerometer data. While their pipeline incorporated variable-length event segmentation, the underlying analytical framework—open-source, R-based, and reliant on post hoc signal processing—aligns closely with the method adopted in our study. Their validation of the GENEAcount algorithm, which showed excellent agreement with activPAL for total daily steps (concordance correlation coefficient = 0.88), supports the potential for low-complexity approaches to yield reliable step count data when appropriately implemented. While our method intentionally reduced the complexity of data interaction, this likely limited the algorithmic sophistication of step detection and contributed to the observed inaccuracies in the walking conditions. We acknowledge that more advanced signal processing techniques—such as machine learning classifiers—have shown high reliability for step detection using GENEActiv data [2]. Therefore, variability in step count accuracy across studies reflects not only device characteristics but also the diversity in analytical pipelines. These findings underscore the need for greater standardisation and validation of processing methods and highlight the trade-off between end user accessibility and analytical precision in clinical research workflows [40]. ~~To improve accessibility, particularly in clinical settings, the chosen approach for analysing GENEActiv data aimed to minimise user interaction, allowing for more automated step count processing. However, this automation likely contributed to the observed inaccuracies, underscoring the need for thorough validation when implementing automated processing techniques.~~

3. *Unclear randomization protocol for device-body placement: The manuscript mentions rotating devices across the arm, waist, and leg, but does not clarify whether a consistent or randomized sequence was used for device positioning across participants. If the order was fixed or unbalanced, it may introduce systematic bias due to fatigue or acclimation effects. Details about the randomization or counterbalancing strategy are necessary to validate the internal control of the experimental design.*

We thank the reviewer for raising this important point and apologise for the lack of clarity in the original description. To minimise systematic bias due to fatigue or acclimation, we used a randomised and counterbalanced protocol for device placement across walking blocks and participants. Device–position combinations were randomised and counterbalanced across participants using built-in functions in Microsoft Excel to ensure full coverage and minimise repetition or order effects. We have updated the Methods section to clarify this randomisation strategy and improve transparency around the internal control of the experimental design.

Data Collection sub-section, page 6, changes from ~Line 128:

*Wearables:* During each five-minute walking block, two devices were worn on the wrists (one per side), two on the ankles, and two on the waist. These positions were categorised as arm, leg, and waist, respectively, with a strap securing the waist devices. After each block, devices were rotated to new positions (e.g., arm to waist, waist to leg, and leg to arm), ensuring all position and gait pattern combinations were tested for every device. Across 12 walking blocks (six slow walk, six shuffle walk), devices were systematically rotated so that each occupied all three positions under both gait conditions. Device–position assignments were randomised and counterbalanced across participants using built-in Microsoft Excel functions, ensuring each device was tested once per gait style at each position while minimising potential order effects.

4. *Insufficient constraint on shuffling gait standardization: The “shuffle walk” condition is described as reduced hip and knee flexion, but no objective control or instrumentation (e.g., motion capture thresholds, wearable inertial measures) was reported to enforce consistency. Since shuffling can vary widely across individuals, lack of constraint may introduce significant variability in motion amplitude and step detection, affecting device comparability and statistical power. More standardized control (e.g., minimum flexion angles or visual feedback) is needed to ensure reproducibility.*

We thank the reviewer for this observation and acknowledge that the shuffling gait condition was not instrumented using motion capture or inertial measurement systems to enforce strict kinematic thresholds. Instead, shuffling was operationally defined as walking with deliberately reduced hip and knee flexion, and participants were provided with standardised verbal instructions and a physical demonstration prior to each trial. This approach was selected to simulate the reduced amplitude in the swing phase of gait patterns often observed in clinical populations, while maintaining a simple and accessible protocol. We recognise that this may introduce variability in movement arcs, particularly at the ankles. However, this variability, which also relates to the diverse participant anthropometry and movement patterns was intentional to better reflect challenges with wearables in a non-specialised environment, where real-time joint angle measures are highly unlikely to be available. Our aim was to assess device performance under realistic, unconstrained movement conditions that reflect how wearables might be used in clinical settings. In this way, the absence of strict biomechanical control enhances the external validity of the experiment, allowing findings to be more generalisable to real-world applications. Additionally, device–position combinations were randomised and counterbalanced as noted above, to minimise the impact of individual variability on outcome comparisons. We agree that future studies incorporating objective constraints (e.g., minimum flexion thresholds) to improve reproducibility or incorporate motion feedback systems to measure arcs of movement to allow adjustment of the statistical analysis, and thus, we have now acknowledged this methodological limitation in the revised manuscript.

Strengths and Limitations sub-section, page 21, changes from ~ Line 498:

An additional limitation of this study is the lack of objective constraint on the shuffling gait condition. Participants were given standardised verbal instructions and a physical demonstration to guide performance. However, the study, conducted in clinical area, did not have access to motion capture or inertial sensors which could have provided data to monitor or enforce specific joint or movement parameters. This, with the diverse anthropometry of the participants, may have introduced variability in gait characteristics, potentially affecting step detection accuracy. However, there was an intentional trade-off to enhance the ecological validity of the study, as our aim was to evaluate device performance under conditions that reflect real-world clinical environments. Future studies may benefit from incorporating objective measures or biofeedback to improve reproducibility of research conditions, while maintaining relevance to clinical populations.

5. *Statistical modelling of repeated measures: The study includes repeated walking blocks and multiple device placements per participant, yet the manuscript does not specify whether statistical models (e.g., linear mixed models) were used to account for within-subject dependency. Failing to model repeated measures appropriately could inflate type I errors or underestimate variance, affecting interpretation of device performance rankings.*

Thank you to the reviewer for this pertinent comment and we apologise for our oversight. We have updated the statistical analysis accordingly using linear mixed-effects models (LMMs). We fit LMMs using the lmer() function from the lme4 package in R, with Step Difference (estimated steps minus true steps) as the outcome variable. The model included fixed effects for Device, Position, and Activity, along with their interactions, and a random intercept for Participant to account for within-subject dependency. To compare devices, we introduced estimated marginal means using the emmeans package and performed pairwise comparisons, followed by non-parametric bootstrapping to derive 95% confidence intervals. The analysis showed that the Apple Watch consistently outperformed the other devices, demonstrating closer alignment with manually counted step scores and significantly differing from the Garmin Vivofit, Fitbit Charge, and Samsung Active. This comparison, now depicted in the additional Figure 8, focused on the four devices with the lowest mean absolute percentage error (MAPE), among which the Apple Watch showed the smallest average deviation from the manual standard. Its superior MAPE performance reinforces the stronger agreement observed in the model. In contrast, the Garmin, Fitbit, and Samsung devices tended to underestimate step counts relative to both the Apple Watch and the manually recorded values. We have added this analysis and a new figure as noted above, to the manuscript and revised the Methods and Results sections accordingly.

Data Analysis sub-section, Page 8, changes from ~ Line 194:

To further investigate differences between devices while accounting for repeated measures within participants, a linear mixed-effects models (LMMs) was deployed. The outcome variable was step count difference (estimated minus true steps), with fixed effects for Device, Position, and Activity, and a random intercept for Participant to account for within-subject dependencies within the data. Estimated marginal means were used to derive pairwise comparisons between devices, and this procedure was bootstrapped (1,000 resamples) to generate robust 95% confidence intervals for pairwise differences. This comparison analysis was restricted to the four devices with the lowest MAPE values—Apple Watch, Garmin Vivofit, Fitbit Charge, and Samsung Active—which represented the top performers in earlier error-based evaluations. Data preprocessing, analysis, and visualisation were performed using the following R packages: dplyr [27], tidyr [28], psych [29], ggplot2 [30], RColorBrewer [31], scales [32], reshape2 [33], lmer [3], emmeans [4], and boot [5].

Results, page 13, changes from ~ Line 306:

Pairwise comparisons of estimated marginal means from the linear mixed-effects model revealed that the Apple Watch recorded significantly higher step counts (estimated vs. true steps) than the Garmin Vivofit, Fitbit Charge, and Samsung Active when collapsed across position and walking activity. Mean differences were 46.6 (95% CI: 27.8 to 65.6), 48.2 (95% CI: 26.9 to 67.2), and 28.9 (95% CI: 7.2 to 50.5) steps, respectively. Given the Apple Watch’s previously demonstrated alignment with true step counts (i.e., lowest MAPE), its higher estimated counts relative to the other devices reflect better accuracy—not overestimation, but a closer match to the manual standard. In contrast, the Garmin, Fitbit, and Samsung devices consistently underestimated step counts. The lack of significant differences among these three (with all pairwise confidence intervals crossing zero) suggests they performed similarly and less accurately than the Apple Watch. Collectively, these results indicate that the Apple Watch provides a significantly more accurate estimate of step count, while the other devices tend to underestimate steps and perform comparably to each other.

Figure 8. Bootstrapped pairwise comparisons of step count differences between activity devices.
Mean differences (estimated minus manual steps) and 95% confidence intervals are shown for the four devices with the lowest MAPE (Apple Watch, Garmin Vivofit, Fitbit Charge, and Samsung Active). The analysis assessed overall device performance irrespective of body position while accounting for within-subject dependency. Confidence intervals that do not cross zero indicate significant differences.

Discussion, page 18, changes from ~ Line 436:

This pattern held across pairwise comparisons, with no significant differences in underestimation among the Garmin Vivofit, Fitbit Charge, and Samsung Active—all of which were significantly less accurate than the Apple Watch. These four devices were selected based on having the lowest mean absolute percentage error (MAPE) scores. Collectively, these findings highlight a shared limitation among the non-Apple devices in detecting steps under slow or irregular walking conditions.

Added References:

1.	Schoenfelder A, Metcalf B, Langford J, Stathi A, Western MJ, Hillsdon M. The analytical and clinical validity of the pfSTEP digital biomarker of the susceptibility/risk of declining physical function in community-dwelling older adults. Sensors. 2023;23(11):5122.

2.	Pavey TG, Gilson ND, Gomersall SR, Clark B, Trost SG. Field evaluation of a random forest activity classifier for wrist-worn accelerometer data. Journal of Science and Medicine in Sport. 2017;20(1):75-80. doi: <https://doi.org/10.1016/j.jsams.2016.06.003>.

3.	Bates D, Mächler M, Bolker B, Walker S. Fitting Linear Mixed-Effects Models Using lme4. Journal of Statistical Software. 2015;67(1):1 - 48. doi: 10.18637/jss.v067.i01.

4.	Lenth R. _emmeans: Estimated Marginal Means, aka Least-Squares Means_. R package version 1.8.2 ed2022.

5.	Davison AC, Hinkley DV. Bootstrap methods and their application: Cambridge university press; 1997.
